# Association between personality traits and smoking cessation among Chinese adults

**DOI:** 10.1186/s40359-023-01442-6

**Published:** 2023-11-17

**Authors:** Weiyun Jin, Bensong Xian, Longlong Zhao, Changle Li

**Affiliations:** 1 College of Humanities Education, Inner Mongolia Medical University, Hohhot, China; 2 School of Health Management, Inner Mongolia Medical University, Hohhot, China; 3 School of Health Management, Fujian Medical University, Fuzhou, 350122 Fujian China

**Keywords:** Personality traits, Big five, Smoking cessation, Sex, Chinese adults, CFPS

## Abstract

**Background:**

Although the tobacco epidemic is one of the greatest public health threats, the smoking cessation rate among Chinese adults is considerably lower. Personality information may indicate which treatments or interventions are more likely to be effective. China is the largest producer and consumer of tobacco worldwide. However, little is known about the association between smoking cessation and personality traits in China.

**Aim:**

This study aimed to examine the association between successful smoking cessation and personality traits among Chinese adults.

**Methods:**

This cross-sectional study used data from the 2018 China Family Panel Studies. Probit regression models were employed to analyze the association between successful smoking cessation and personality traits stratified by sex.

**Results:**

Lower scores for neuroticism (Coef.=-0.055, *p* < 0.1), lower scores for extraversion (Coef.=-0.077, *p* < 0.05), and higher scores for openness to experience (Coef.=0.045, *p* < 0.1) predicted being a successful male quitter after adjusting for demographics. Moreover, lower scores for conscientiousness (Coef.=-0.150, *p* < 0.1) predicted being a successful female quitter after adjusting for demographics.

**Conclusion:**

The empirical findings suggested that among Chinese men, lower levels of neuroticism, lower levels of extraversion, and higher levels of openness to experience were associated with a higher likelihood of smoking cessation. Moreover, lower levels of conscientiousness were associated with successful smoking cessation among Chinese women. These results showed that personality information should be included in smoking cessation interventions.

## Introduction

The tobacco epidemic is one of the greatest public health threats, resulting in 7.69 million deaths a year worldwide [1]. In China, 26.6% of adults aged 15 years or older were current smokers in 2018. Men (50.5%) were more likely than women (2.1%) to be current smokers [2]. Although smoking has been proven to be a significant cause of diseases such as cancers, heart diseases, stroke, lung diseases, and chronic obstructive pulmonary disease [3], the smoking cessation rate (percentage of former smokers out of current and former smokers) among Chinese adults aged 15 years or older was 20.1%, and the smoking cessation rate of daily smokers was only 15.6% in 2018 [2]. Successful smoking cessation is associated with the amount of tobacco smoking, age, socioeconomic status, marital status, alcohol consumption, disease morbidity, and physiological, psychological, and behavioral factors [4–8]. Psychological factors include personality traits, self-control, depressive disorders, and anxiety [9, 10].

Personality traits reflect basic trait dimensions on which people differ [11]. The most widely accepted taxonomy of personality traits is the five-factor model of personality (FFM) known as the ‘Big Five’ personality traits. The FFM is a set of five trait dimensions describing most personality traits: neuroticism, extraversion, openness to experience, agreeableness, and conscientiousness [12]. Identification of personality traits associated with smoking initiation and successful smoking cessation may help develop tailored smoking cessation programs. The effects of personality traits on smoking initiation were similar across many studies. For example, Van Loon et al. [6], Sallis et al. [13], and Hakulinen et al. [14] found that higher levels of extraversion were positively related to smoking initiation. Hakulinen et al. [14] and Von Ah [15] reported that smoking initiation was associated with lower levels of conscientiousness.

The findings are mixed on how personality traits affect smoking cessation. For example, Van Loon et al. [6], Shadel et al. [16], and Berlin and Covey [17] found that the association between smoking cessation and personality traits was not confirmed. Hakulinen et al. [14] and Cosci et al. [18] reported that higher levels of neuroticism were negatively related to smoking cessation. Abe et al. [19] and Zvolensky et al. [20] showed that higher levels of conscientiousness were associated with smoking cessation. However, Lee et al. [21] reported the opposite; lower levels of conscientiousness were related to smoking cessation among women. Leung et al. [22] found that people with lower levels of openness to experience had a greater likelihood of quitting smoking. Stephan et al. [23] found that quitters were more likely to have higher levels of agreeableness. Gainforth et al. [24] reported that people with higher levels of extraversion had greater odds of being quitters.

The extremely low prevalence of smoking among women in China has been mainly attributed to social norms against women smoking [25]. However, women are more likely than men to have a higher risk of smoking-related morbidity and mortality and face gender-specific barriers to smoking cessation [26, 27]. Personality traits show apparent gender differences. For example, women tend to score higher than men in agreeableness, meaning women are more nurturing, tenderminded, and altruistic than men [28, 29]. Therefore, we should consider different personality patterns in male and female smokers seeking effective methods for successful smoking cessation [30].

Past studies provide substantial evidence that smoking initiation is associated with different personality traits. Some personality traits are protective factors for smoking initiation, and others are risk factors [31]. However, evidence on the association between smoking cessation and personality traits is limited. Moreover, although the association between smoking cessation and personality traits has been documented, thus far, only a few studies have analyzed this phenomenon using a nationally representative dataset. Last, as the world’s largest producer and consumer of tobacco, little is known about the association between smoking cessation and personality traits in China. The previous findings may not be applicable to the Chinese population. To fill these research gaps, the objective of this study was to examine the association between successful smoking cessation and personality traits among Chinese adults using a nationally representative dataset.

## Methods

### Theoretical model

**Fig. 1:**
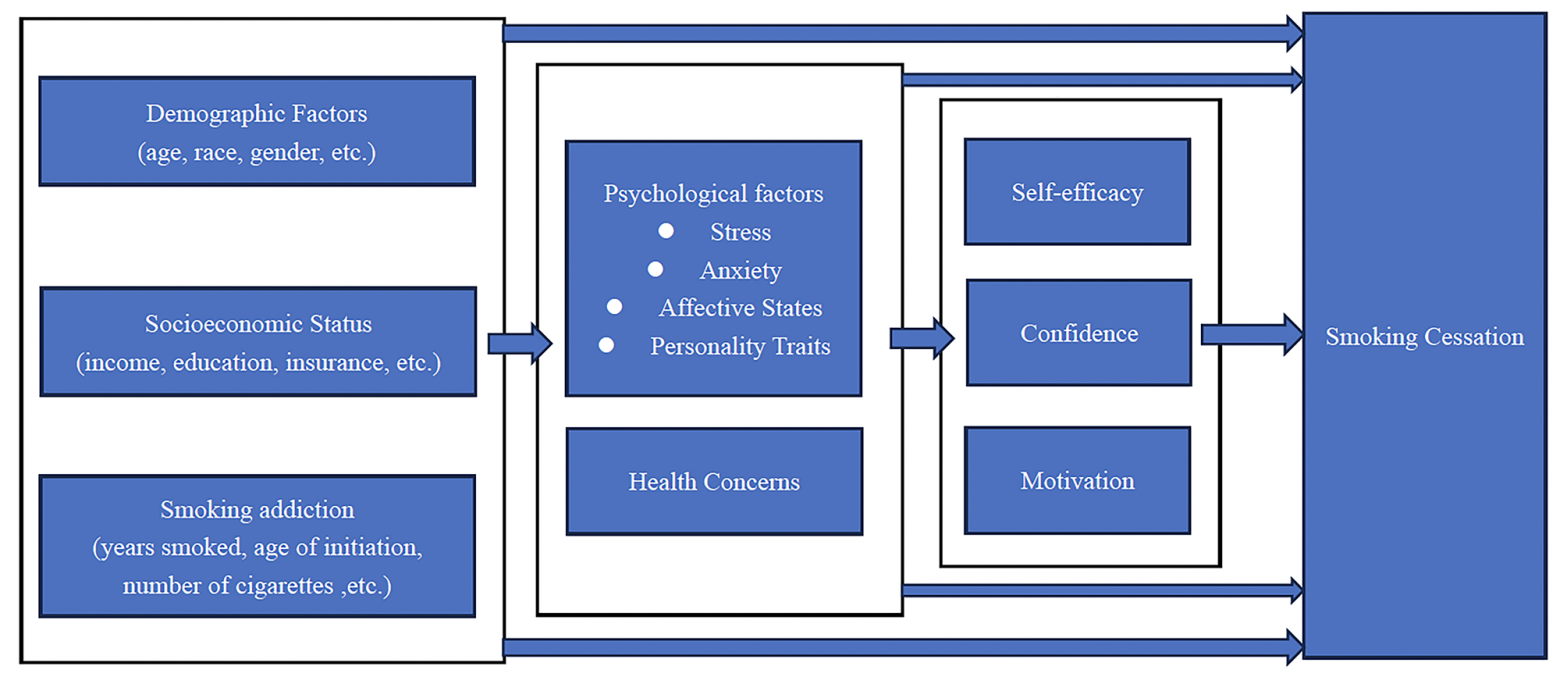
Conceptual framework to identify relevant factors affecting smoking cessation

A theoretical path model of smoking cessation guided the current study [32]. The framework helps identification of potential factors affecting success in quitting smoking. A conceptual framework has been presented in Fig. 1. Demographic factors and socioeconomic status may affect smoking cessation interventions, although their effects are not in any consistent way [32, 33]. Demographic factors and socioeconomic status also significantly affect both psychological factors and health concerns [32, 34]. Psychological factors and health concerns can predict motivation, self-efficacy, and confidence to quit and also directly affect smoking cessation [32]. Abstinence motivation, self-efficacy, and confidence have been shown to play significant roles in smoking cessation [32]. It should be noted that some of the variables in the conceptual model may not be available in the CFPS. Still, this generalized framework will be helpful to ensure that all relevant variables are considered in the empirical analysis.

### Data sources

**Fig. 2:**
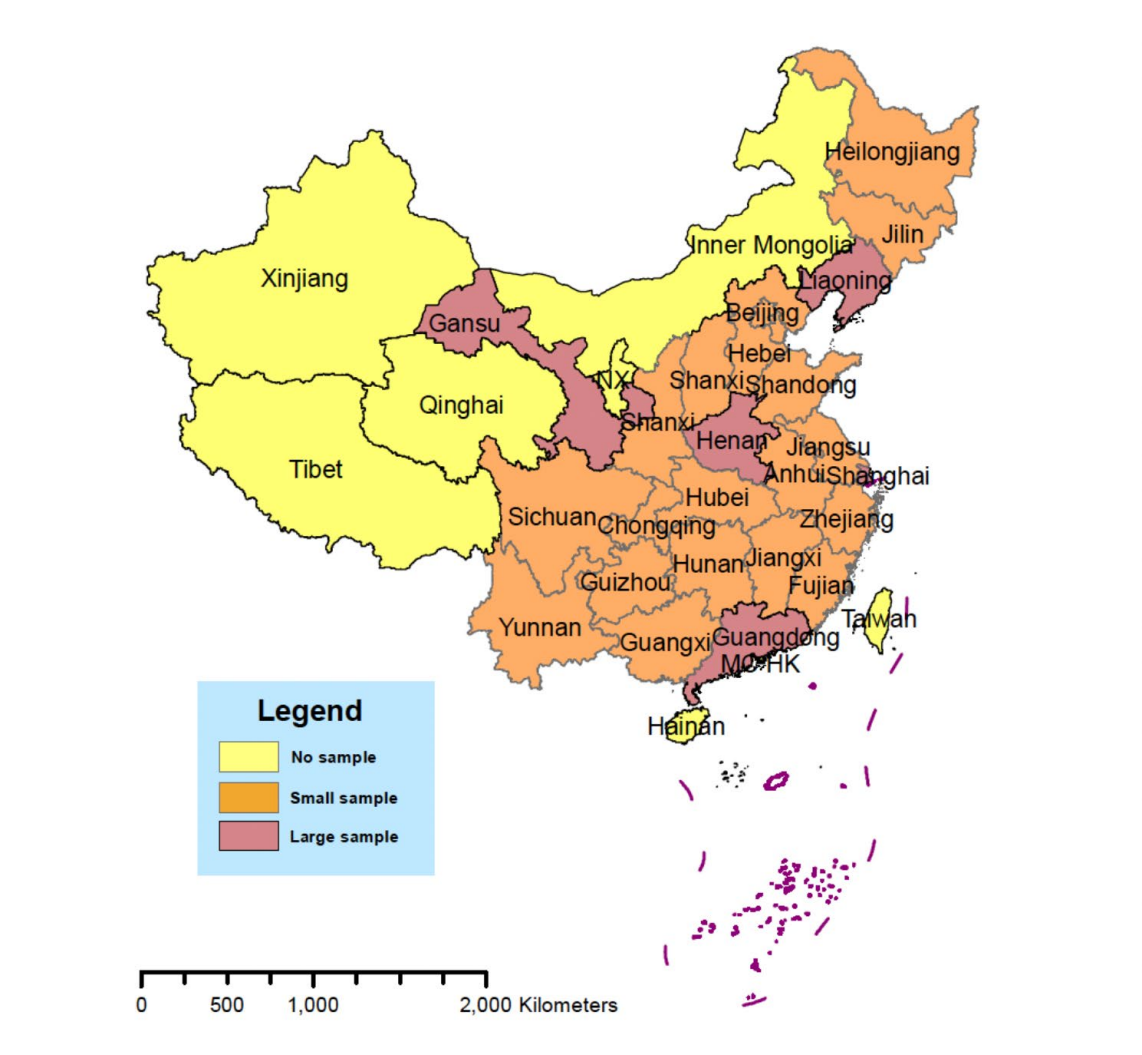
The representativeness of the samples

The data used in this study were obtained from the 2018 China Family Panel Studies (CFPS) initiated by the Institute of Social Science Survey of Peking University in 2010. The CFPS is a general-purpose, nationally representative, longitudinal survey. A multistage probability sample proportional to size was used to select and interview households covering 25 provinces and their administrative equivalents. Five provinces or administrative equivalents (Liaoning, Shanghai, Henan, Guangdong, and Gansu) were selected to oversample populations, and the remaining twenty provinces or administrative equivalents were grouped (see Fig. 2). Each of the six CFPS subsamples was selected through three stages. The CFPS drew counties or their administrative equivalents in the first stage and then drew villages (rural areas) or resident committees (urban areas) in selected counties/districts in the second stage. Finally, households were drawn from a selected village or resident committee in the third stage. The CFPS represents 95% of the total population in the Chinese mainland [35].

The CFPS employed multi-module designs for questionnaires. Each questionnaire consisted of different modules regarding the specific situations of the individuals and households interviewed. The CFPS questionnaires include questions on demographic background, family structure/transfer, health status and physical functioning, health care utilization, insurance status, work, income, expenditure, asset ownership, community-level information, etc. The CFPS primarily conducts face-to-face interviews. When the CFPS fails to complete face-to-face interviews, telephone or web-based interviews are used as a substitute. More details on the sampling and data collection process are available in Xie and Hu [36].

The CFPS respondents are reinterviewed every two years, with the first wave in 2010 and five follow-ups in 2012, 2014, 2016, 2018, and 2020. The 2018 survey included 30,593 adults (aged ≥ 16 years) who answered the survey questionnaire. From the full sample, only adults (aged ≥ 16 years) who reported former smoking or current smoking were selected (9,515 adults). After eliminating all cases with missing relevant data (173 adults), the final sample consisted of a total of 9,342 adults.

#### Measures

##### Successful Smoking cessation

Most studies on successful smoking cessation were conducted in the USA and Asian countries. In these studies, successful smoking cessation was defined as individuals who formerly smoked and stopped smoking at least 6 months, 6 to 12 months, or at least 12 months prior to the interview [37–41]. The current study defined successful smoking cessation as former smokers who stopped smoking cigarettes for at least 12 months. In the CFPS, each respondent was asked, ‘Have you ever smoked?’. Respondents who answered ‘Yes’ were then asked, ‘How old were you when you totally stopped smoking completely?’. The outcome variable, successful smoking cessation, was coded as 1 ‘yes’ and 0 ‘no’.

##### Personality traits

This study used the 15-item short version of the Big Five Inventory (BFI-S) to measure personality traits. In this inventory, three items assess each of the Big Five dimensions (neuroticism, extraversion, openness to experience, agreeableness, and conscientiousness). Each dimension includes positively and negatively keyed items. Items are rated on a 5-point Likert scale, ranging from 1 (strongly disagree) to 5 (strongly agree). After reverse-scoring the negatively keyed items and averaging the scores of each dimension, the score of each personality trait was calculated, and higher scores indicated a higher level of that trait. The BFI-S has been proven to be easy to administer, valid, and reliable [42].

##### Control variables

The analysis considered the following four categories of variables as control variables: (1) smoking addiction (age of smoking initiation), (2) demographic factors (age), (3) socioeconomic status (place of residence, educational attainment, marital status, medical insurance coverage, household income), and (4) health concerns (proxy indicators using self-rated health status and chronic conditions). Definitions of all the relevant variables are listed in Table 1.

**Table 1:** Definitions of variables (N = 9,342)

Variable	Description	Mean (SD)	%
Successful smoking cessation	1 if the individual was smoke free for at least 12 months; 0 otherwise		7.10
Personality traits			
Neuroticism	Average of the scores for neuroticism, ranging from 1 (strongly disagree) to 5 (strongly agree)	2.86 (0.73)	
Extraversion	Average of the scores for extraversion, ranging from 1 (strongly disagree) to 5 (strongly agree)	3.40 (0.71)	
Openness to experience	Average of the scores for openness to experience, ranging from 1 (strongly disagree) to 5 (strongly agree)	3.22 (0.87)	
Agreeableness	Average of the scores for agreeableness, ranging from 1 (strongly disagree) to 5 (strongly agree)	3.79 (0.62)	
Conscientiousness	Average of the scores for conscientiousness, ranging from 1 (strongly disagree) to 5 (strongly agree)	3.87 (0.66)	
Sex	1 if the individual was male; 0 for female		93.76
Age	Continuous variable, actual age in years	47.59 (0.16)	
Age of smoking initiation	Continuous variable, age at first puff	20.65 (6.59)	
Place of residence	1 if rural resident; 0 for urban resident		50.63
Educational attainment			
Illiterate/Semiliterate	1 if the individual was illiterate or semiliterate; 0 otherwise		17.87
Elementary school	1 if the individual attended elementary school; 0 otherwise		22.04
Middle school	1 if the individual graduated from middle school; 0 otherwise		33.01
High school	1 if the individual graduated from high school; 0 otherwise		17.30
> 3-years of college	1 if the individual completed more than three years of college; 0 otherwise		9.78
Married	1 if the individual was married; 0 otherwise		80.31
No insurance	1 if the individual did not have medical insurance; 0 otherwise		8.27
Household income	Continuous variable, net household income (×10,000 yuan)	9.51 (17.65)	
Self-rated health status			
Poor	1 if the individual reported a poor health status; 0 otherwise		13.44
Fair	1 if the individual reported a fair health status; 0 otherwise		12.55
Good	1 if the individual reported an excellent, very good, or good health status; 0 otherwise		74.01
Chronic conditions	1 if the individual had doctor-diagnosed chronic diseases in the past six months; 0 otherwise		13.98

##### Data Analysis

A descriptive analysis of successful smoking cessation was performed by considering various individual characteristics and personality traits. Statistical significance between groups was assessed through Pearson’s chi-square test for categorical variables and Student’s t test for continuous variables.

Since the dependent variable was a binary response variable (successful smoking cessation), probit regression models were used to analyze the association of successful smoking cessation and personality traits stratified by sex. Because of different personality patterns among males and females, stratification was essential to avoid potential bias created by sex differences. The Model I was unadjusted. Age is a predictor of quit attempts and quit success in smoking cessation [43]. The control variables of adjusted models were demographic factors (Model II) and demographic factors, socioeconomic status, smoking addiction, and health concerns (Model III). The results are presented as coefficients (Coef.) along with their standard errors (SEs). All statistical analyses were conducted using Stata version 17 (StataCorp, College Station, TX).

## Results

A descriptive summary of all variables is shown in Table 1. The total sample size was 9,342; 93.76% of respondents were male, and the mean age was approximately 47 years. Approximately 60% of the respondents had at least a middle school education. A total of 7.10% of adult smokers successfully quit smoking for at least 12 months. Table 2 shows the differences between successful quitters and nonquitters according to demographics, social determinants, and health status. The results of univariable analyses indicated that successful quitters differed significantly from nonquitters in terms of their age, sex, residence, educational attainment, marital status, household income, self-rated health status, and chronic conditions.

**Table 2:** Differences between successful quitters and nonquitters according to demographics, social determinants, and health status

	Successful smoking cessation	
	Yes (N = 663)	No (N = 8,679)
Sex (%)***		
Males	85.22	94.41
Females	14.78	5.59
Age group (%)**		
16–29 years	22.78	15.37
30–39 years	14.48	18.17
40–49 years	12.52	19.52
50–59 years	14.63	21.37
60 + years	35.60	25.57
Place of residence (%)***		
Rural	42.23	51.27
Urban	57.77	48.73
Educational attainment (%)***		
Illiterate/Semiliterate	16.89	17.94
Elementary school	19.91	22.20
Middle school	29.71	33.26
High school	21.72	16.96
> 3 years of college	11.76	9.63
Married (%)***		
Yes	73.45	80.84
No	26.55	19.16
No Insurance (%)		
Yes	8.14	8.28
No	91.86	91.72
Household income (%)*		
Low income (in the first quartile)	22.47	25.27
Lower middle income (in the second quartile)	25.19	24.91
Upper middle income (in the third quartile)	23.68	25.19
High income (in the highest quartile)	28.66	24.63
Self-rated health status***		
Poor	17.80	13.11
Fair	12.67	12.54
Good	69.53	74.35
Chronic conditions***		
Yes	21.72	13.39
No	78.28	86.61

Note: Pearson’s chi-square test, ***significantly different (*P* < 0.01); *significantly different (*P* < 0.1)

Table 3 shows the differences between successful quitters and nonquitters according to personality traits and age of smoking initiation. Successful quitters had lower scores for extraversion (3.34 ± 0.70 vs. 3.40 ± 0.71; *p* < 0.05) and lower scores for conscientiousness (3.82 ± 0.72 vs. 3.87 ± 0.65, *p* < 0.05).

**Table 3:** Differences between successful quitters and nonquitters according to personality traits and age of smoking initiation

	Successful smoking cessation	
	Yes (N = 663)	No (N = 8,679)
Personality traits		
Neuroticism (mean ± SD)	2.85 ± 0.75	2.86 ± 0.73
Extraversion (mean ± SD)**	3.34 ± 0.70	3.40 ± 0.71
Openness to experience (mean ± SD)	3.22 ± 0.90	3.22 ± 0.87
Agreeableness (mean ± SD)	3.77 ± 0.62	3.79 ± 0.62
Conscientiousness (mean ± SD)**	3.82 ± 0.72	3.87 ± 0.65
Age of smoking initiation (mean ± SD)	20.26 ± 6.79	20.69 ± 6.57

Note: Two-sample t test, **significantly different (*P* < 0.05)

Table 4 shows successful smoking cessation associated with personality traits among Chinese men using probit models. In Model I, the coefficients of personality traits were not adjusted. Lower scores for neuroticism (Coef.=-0.055, *p* < 0.1) and lower scores for extraversion (Coef.=-0.075, *p* < 0.05) predicted being a successful male quitter. In Model II, the coefficients of personality traits were adjusted for demographics. Lower scores for neuroticism (Coef.=-0.055, *p* < 0.1), lower scores for extraversion (Coef.=-0.077, *p* < 0.05), and higher scores for openness to experience (Coef.=0.045, *p* < 0.1) predicted being a successful male quitter. In Model III the coefficients of personality traits were adjusted for demographics, social determinants, smoking addiction, and health concerns. Lower scores for extraversion (Coef.=-0.073, *p* < 0.05) predicted being a successful male quitter. Table5 shows the successful smoking cessation associated with personality traits among Chinese women using probit models. In Model I, the coefficients of personality traits were not adjusted. Lower scores for conscientiousness (Coef.=-0.167, *p* < 0.1) predicted being a successful female quitter. The Model II results were very similar to the Model I.

**Table 4:** Probit regression results for factors associated with successful smoking cessation among Chinese men (N = 8,759)

	Model I	Model II	Model III
	Coef. (SE)	Coef. (SE)	Coef. (SE)
**Personality traits**			
Neuroticism	-0.055 (0.030)*	-0.055 (0.030)*	-0.044 (0.030)
Extraversion	-0.075 (0.031)**	-0.077 (0.031)**	-0.073 (0.032)**
Openness to experience	0.037 (0.026)	0.045 (0.026)*	0.028 (0.026)
Agreeableness	-0.027 (0.036)	-0.031 (0.036)	-0.049 (0.037)
Conscientiousness	-0.028 (0.034)	-0.039 (0.035)	-0.008 (0.035)
**Demographics**			
Age		0.003 (0.001)**	0.007 (0.002)***
**Social determinants**			
Place of residence			-0.112 (0.045)**
Educational attainment			
Illiterate/Semiliterate (ref.)			
Elementary school			0.079 (0.074)
Middle school			0.130 (0.072)*
High school			0.319 (0.079)***
> 3 years of college			0.315 (0.095)***
Married			-0.249 (0.055)***
No insurance			-0.030 (0.079)
Household income			-0.001 (0.002)
**Health status**			
Self-rated health status			
Poor			0.055 (0.083)
Fair (ref.)			
Good			-0.008 (0.066)
Chronic conditions			0.290 (0.060)***
**Age of smoking initiation**			-0.018 (0.004)***
Constant	-1.019 (0.209)***	-1.107 (0.213)***	-0.955 (0.244)***

Note: Asterisks^***^ indicate statistical significance at the 1% level, ^**^ at the 5% level, and ^*^at the 10% level

**Table 5:** Probit regression results for factors associated with successful smoking cessation among Chinese women (N = 583)

	Model I	Model II	Model III
	Coef. (SE)	Coef. (SE)	Coef. (SE)
**Personality traits**			
Neuroticism	-0.015 (0.083)	-0.016 (0.083)	0.010 (0.086)
Extraversion	-0.032 (0.088)	-0.032 (0.088)	-0.060 (0.092)
Openness to experience	0.027 (0.066)	0.013 (0.068)	0.021 (0.072)
Agreeableness	-0.055 (0.096)	-0.038 (0.097)	-0.082 (0.103)
Conscientiousness	-0.167 (0.089)*	-0.150 (0.091)*	-0.098 (0.096)
**Demographics**			
Age		-0.003 (0.003)	0.002 (0.005)
**Social determinants**			
Place of residence			-0.256 (0.140)*
Educational attainment			
Illiterate/Semiliterate (ref.)			
Elementary school			0.087 (0.191)
Middle school			0.205 (0.205)
High school			0.212 (0.269)
> 3 years of college			0.103 (0.336)
Married			-0.123 (0.142)
No insurance			-0.328 (0.213)
Household income			0.017 (0.007)**
**Health status**			
Self-rated health status			
Poor			0.051 (0.226)
Fair (ref.)			
Good			0.073 (0.204)
Chronic conditions			0.022 (0.168)
**Age of smoking initiation**			-0.008 (0.006)
Constant	-0.040 (0.569)	0.054 (0.579)	-0.132 (0.705)

Note: Asterisks^***^ indicate statistical significance at the 1% level, ^**^ at the 5% level, and ^*^at the 10% level

## Discussion

This study examined the association between successful smoking cessation and personality traits among Chinese adults. Data from a nationally representative survey were used for the analysis. The results indicated that only approximately 7% of adult smokers successfully quit for 12 months, which was consistent with the findings in the USA [44]. The adult smoking cessation rate is very low in China. Approximately 90% of adult smokers who have tried to stop smoking in the past 12 months have never received quitting assistance [2], which implies that inadequate smoking cessation programs may lead to a low cessation rate in China. Various national or international clinical guidelines suggest that smoking cessation programs include behavioral support and pharmacological treatments [45]. Knowledge regarding personality traits should be taken into consideration for individually tailored smoking cessation programs, which could lead to both improved uptake and efficacy [13]. The Chinese government needs to develop smoking cessation programs that are tailored to the needs of adult smokers. Therefore, identifying the association between successful smoking cessation and personality traits will be of interest to physicians and policymakers.

We have used probit regression models to examine the association between successful smoking cessation and personality traits. The results indicated that lower levels of neuroticism were associated with a higher likelihood of smoking cessation among Chinese men. However, the association became nonsignificant after adjusting for demographic factors, socioeconomic status, smoking addiction, and health concerns. Similar results have been obtained confirming the association between levels of neuroticism and smoking cessation. A cross-sectional and longitudinal individual-participant meta-analysis has shown that smoking cessation was negatively associated with neuroticism [14]. In addition, a cross-sectional study found that male smokers were much more neurotic than men who stopped smoking [46]. Neuroticism is the personality trait tendency toward anxiety, anger, self-consciousness, irritability, emotional instability, and depression [47]. An array of tobacco withdrawal symptoms accompanies smoking cessation, and these symptoms may be experienced more intensely by smokers with higher levels of neuroticism [14]. Moreover, smokers with higher levels of neuroticism respond poorly to environmental stress [48], which may increase the likelihood of smoking relapse.

We found that among Chinese men, lower levels of extraversion predicted being a successful quitter. This association was maintained after adjusting for demographic factors, socioeconomic status, smoking addiction, and health concerns. Our finding is consistent with previous Poland-based research. The cross-sectional study found that successful quitters’ facets of extraversion score were lower than current smokers [49]. However, another correlational study in the UK reported the opposite finding [24]. Extraversion is a personality trait characterized by sociability, assertiveness, high activity levels, and impulsivity [50]. Smoking is considered a highly acceptable social activity and a part of social interaction in China, especially among males [51]. The essential feature of extraversion is the disposition to enjoy social situations [52]. Therefore, cigarette refusal among smokers with higher levels of extraversion is more difficult in social situations, which may decrease the likelihood of smoking cessation.

Higher levels of openness to experience were positively associated with successful smoking cessation among Chinese men after adjusting for demographic factors. In contrast, a 7-year cohort study showed that Chinese smokers with lower levels of openness to experience had a greater likelihood of quitting smoking [22]. However, the association became nonsignificant after adjusting for demographic factors, socioeconomic status, smoking addiction, and health concerns. Individuals with higher levels of openness to experience are willing to try new things and embrace changes in their lives [53]. Although becoming a successful quitter is a challenge for smokers, smokers with higher levels of openness to experience are more likely to embrace the opportunity to remove smoking behavior from their lives and become a different version of themselves [54]. Moreover, smoking cessation services and medications are still relatively new in China. Smokers with higher levels of openness to experience are more likely to use these services and medications, which can increase quitting success rates.

Lower levels of conscientiousness were associated with successful smoking cessation among Chinese women, which was consistent with the findings in Japan [21]. However, the association became nonsignificant after adjusting for demographic factors, socioeconomic status, smoking addiction, and health concerns. Female sex was associated with an increased likelihood of experiencing depression [55]. Moreover, lower levels of conscientiousness have been found to be associated with an increased risk of depression and other mental disorders [56]. Therefore, female smokers with lower levels of conscientiousness who experience a worse mental health status are more likely to be successful quitters.

### Limitations

Although the current study employed a nationally representative dataset to analyze the association between successful smoking cessation and personality traits among Chinese adults, several limitations should be emphasized. First, this was a cross-sectional study based on the 2018 CFPS. It had limitations in providing causal inferences. Further longitudinal studies should be performed to track changes in smoking behavior and personality traits over time and may provide more robust evidence for the direction and mechanism of the association. Although the CFPS is a biennial longitudinal survey, the CFPS survey only collects information on personality traits in 2018. Second, the data were obtained via face-to-face or telephone interviews, and thus, limitations of all self-reported data exist, which may be subject to bias and measurement error. For example, participants may under-report their smoking behavior or over-report their success with stopping smoking, leading to inaccurate estimates of the association between successful smoking cessation and personality traits. Last, although this study adjusted for a wide variety of covariates, it is possible that unknown or unmeasured confounders may explain the current findings.

## Conclusion

The purpose of this study was to identify the association between successful smoking cessation and personality traits among Chinese adults. The empirical findings suggested that among Chinese men, lower levels of neuroticism, lower levels of extraversion, and higher levels of openness to experience predicted being a successful quitter. Moreover, lower levels of conscientiousness were associated with successful smoking cessation among Chinese women. Nevertheless, the association between successful smoking cessation and personality traits among Chinese adults was not straightforward and may have varied according to sex. These results showed that personality information should be incorporated into smoking cessation interventions. The heterogeneity of smokers on several dimensions has been documented [57]. Personality information may indicate which treatments or interventions are more likely to be effective [58]. Increased attention and smoking cessation support for male smokers with higher levels of neuroticism and extraversion and encouraging the use of smoking cessation services and medications for male smokers with higher levels of openness to experience can improve the outcome of smoking cessation interventions. Female smokers with lower levels of conscientiousness have increased odds of suffering from depression, and smoking cessation interventions could effectively reduce female smokers’ risk of depression.

## Data Availability

The dataset used for drafting the paper is a publicly available dataset available in the Peking University Open Research Data Platform repository. The dataset is downloadable for research purposes through the link: https://opendata.pku.edu.cn/dataset.xhtml?persistentId=doi:10.18170/DVN/45LCSO.
